# First Investigative Study of Azole-Resistant *Aspergillus fumigatus* in the Environment in Burkina Faso

**DOI:** 10.3390/ijerph18052250

**Published:** 2021-02-25

**Authors:** Isidore W. Yerbanga, Agustin Resendiz-Sharpe, Sanata Bamba, Katrien Lagrou, Seydou Nakanabo Diallo, Hector Rodriguez-Villalobos, Olivier Denis, Isabel Montesinos

**Affiliations:** 1 Ecoles Doctorales des Sciences de la Santé, Université Nazi Boni, Bobo-Dioulasso, 01 BP 1091 Bobo-Dioulasso 01, Burkina Faso; hsanata@yahoo.fr (S.B.); naksaid2006@yahoo.fr (S.N.D.); 2 Service de laboratoire, Centre Hospitalier Universitaire Régional de Ouahigouya, Ouahigouya, 01 BP 36 Ouahigouya 01, Burkina Faso; 3 Department of Microbiology, Immunology and Transplantation, KU Leuven, University Hospitals Leuven, Leuven, Herestraat 49-Box 1030, 3000 Leuven, Belgium; agustin.resendizsharpe@kuleuven.be (A.R.-S.); katrien.lagrou@uzleuven.be (K.L.); 4 Département des laboratoires, Centre Hospitalier Universitaire Sourô Sanou, Bobo-Dioulasso, 01 BP 676 Bobo-Dioulasso 01, Burkina Faso; 5 Department of Laboratory Medicine and National Reference Center for Mycosis, Excellence Center for Medical Mycology (ECMM), University Hospitals Leuven, Herestraat 49-Box 1030, 3000 Leuven, Belgium; 6 Programme de Recherche sur les Maladies Parasitaires et les Vecteurs, Centre Muraz, Institut National de Santé Publique, Bobo-Dioulasso, 01 BP 390 Bobo-Dioulasso 01, Burkina Faso; 7 Department of Microbiology, Cliniques Universitaires Saint-Luc–Université Catholique de Louvain, Brussels, Avenue Hippocrate 10, 1200 Bruxelles, Belgium; hector.rodriguez@uclouvain.be; 8 Department of Microbiology, CHU Namur Site-Godinne, Université Catholique de Louvain, Brussels, Rue Dr Gaston Therasse 1, 5530 Yvoir, Belgium; odenis@ulb.ac.be; 9 Ecole de Santé Publique, Université Libre de Bruxelles, Brussels, Route de Lennik 808, 1070 Anderlecht, Belgium; 10 Department of Microbiology, LHUB-ULB, Université Libre de Bruxelles, Brussels, Rue Haute 322, 1000 Bruxelles, Belgium; carlota.montesinos@erasme.ulb.ac.be

**Keywords:** azole-resistance, *Aspergillus fumigatus*, *cyp51A* gene, Burkina Faso

## Abstract

Azole-resistant *Aspergillus fumigatus* (ARAF) strains have been reported on all continents, however, limited data exist on these strains in Africa, while several factors, mainly environmental ones, suggest their presence on this continent. This study aimed to assess the environmental prevalence of ARAF strains in Burkina Faso, a country situated in the West African region where data on ARAF is non-existent. In total, 120 environmental samples (soil) were collected and analyzed. Samples were screened for resistance using three azole-containing agar plates; one without azole antifungal (growth control) and two supplemented with either itraconazole (4 mg/L) or voriconazole (2 mg/L). The EUCAST susceptibility testing method was used to confirm the azole-resistant phenotype of *A. fumigatus* sensu-stricto isolates. Mutations in the *cyp51A* gene were determined by sequencing. Of the 120 samples, 51 positive samples showed growth of *A. fumigatus* isolates on control medium. One ARAF (2%; 1/51) isolate was found amongst *A. fumigatus* positive samples and harbored the F46Y/M172V/E427K *cyp51A* mutations. No TR34/L98H or TR46/Y121F/T289A mutations were observed. Our study described the first *A. fumigatus* isolate resistant to an azole antifungal in Burkina Faso.

## 1. Introduction

*Aspergillus fumigatus* is the main species responsible for *Aspergillus*-related diseases, with a mortality rate ranging from 30% to 95% among invasive aspergillosis patients [1]. Azole antifungals are the cornerstone in the treatment of aspergillosis. However, the emergence of azole-resistance in *A. fumigatus* seriously compromises the management of patients suffering from *Aspergillus* diseases [2]. Resistance rates vary between countries and underlying conditions [2,3]. In Africa, only Tanzania and Kenya have performed susceptibility-related studies and reported the presence of Azole-resistant *Aspergillus fumigatus* (ARAF) isolates [4,5,6]. However, there are several arguments supporting the presence of ARAF isolates in other African countries. The widespread use of azole fungicides (14a-demethylase inhibitors; DMIs) in agricultural fields in Africa could favor the selection of azole resistant isolates of *A. fumigatus* with cross-resistance to medical azoles [7,8,9]. Furthermore, the ability of ARAF spores to migrate from one country to another is an additional argument in favor of the presence of these resistant isolates in other African countries [3]. In this cross-sectional study, we sought to assess the environmental prevalence of ARAF in Burkina Faso, a country situated in the West African region where data on ARAF is non-existent.

## 2. Materials and Methods

### 2.1. Soil Samples Analysis (Collection and Processing)

This study was carried out in Bobo-Dioulasso, the second largest city of Burkina Faso. Environmental samples (5 g of soil) were collected in November 2019 (dry season) from several predetermined urban locations (hospital, residential, university, and nurseries) and rural locations (agricultural and forest). In the agricultural sector, soil samples were collected from cotton, rice, and market gardening fields. Laboratory analysis of soil samples were carried out at the department of microbiology, immunology and transplantation in Leuven (Belgium). From each soil sample, 2 g were dissolved in 8 mL solution of 0.85% NaCl + 0.01% Tween 20. After vortexing, 100 microliters were plated into three Sabouraud dextrose agar (SDA) media plates; one without azole antifungal (growth control) and two supplemented with either itraconazole (4 mg/L) or voriconazole (2 mg/L). The samples were then incubated at 50 °C for 72–96 h to restrict the growth of most environmental fungi. The growth control medium was used to determine the total number of *A. fumigatus* isolates. The remaining two agar media plates supplemented with a triazole were used to identify suspected ARAF isolates.

### 2.2. Fungal Identification

Microscopic and macroscopic features of each colony were used for the identification of *A. fumigatus* species. Matrix-assisted laser desorption ionization-time of flight mass spectrophotometry (MALDI-TOF-MS) was used to confirm suspected ARAF isolates that grew on media containing either itraconazole or voriconazole as *A. fumigatus* sensu-stricto as published previously [10]. Briefly, the isolates were gently scrapped with a sterile scalpel and then transferred in a mixture of sterile water and anhydrous ethylic alcohol for protein precipitation. Samples were subsequently treated with 70% formic (5 min incubation) followed by 100% acetonitrile (5 min incubation) for protein extraction. From each isolate, three replicates consisting of 1 μL of supernatant extract was deposited on a spot of the target-plate and covered when dry with 1 μL of the matrix solution. Spectra were determined using the Bruker Biotyper MALDI-TOF MS system (Bruker Daltonics, Bremen, Germany) and identified with the mass spectrometry identification (MSI) platform. We consider an adequate species level identification threshold of 20 or above.

### 2.3. A. fumigatus Susceptibility Testing and cyp51A Gene Sequencing

The European Committee on Antimicrobial Susceptibility Testing (EUCAST) broth microdilution method for susceptibility testing and clinical breakpoints of molds were used to confirm the triazole-resistant phenotype of suspected ARAF isolates [11]. Sequencing of the *cyp51A* gene was performed on azole resistant isolates as published previously [12]. In brief, amplified DNA from selected *A. fumigatus* isolates were sequenced using specifically designed primers for the *cyp51A* gene of *A. fumigatus* and the BigDye-Terminator-v3.1 cycle-sequencing kit (Applied-Biosystems, Vilnius, Lithuania). Reaction products were purified (DyeEX2.0-Kit; Qiagen, Hilden, Germany), dried, and reconstituted in 20 µL of HiDi Formamide (Applied Biosystems, Paisley, UK) according to the manufacturer’s protocol. Reconstituted products were run on an ABI3730xl Genetic Analyzer (Applied-Biosystems, Foster City, CA, USA). Obtained sequences were aligned to create a consensus sequence using the CLC-Genomics-Workbench software (CLC-bio, Aarhus, Denmark). Consensus sequences were compared to the *A. fumigatus cyp51A* gene reference sequence ATCC366071.

### 2.4. Data Analysis

Isolates proportions and table were done using Microsoft office excel 2013 software.

## 3. Results

Table 1 summarizes the characteristics and distribution of analyzed samples by environmental site. Of the 120 samples, 51 samples were positives for the growth of *A. fumigatus* in control medium (646 *A. fumigatus* isolates in total). From this two samples grew, each one isolate on SDA supplemented with voriconazole. Amongst these two isolates, one isolate (2%, 1/51), from an urban location, was confirmed as resistant to voriconazole (Minimum Inhibitory Concentration: MIC 2 mg/L), isavuconazole (MIC = 4 mg/L), and posaconazole (MIC = 0.5 mg/L), but susceptible to itraconazole (MIC = 1 mg/L). Sequencing of the *cyp51A* gene identified the F46Y/M172V/E427K mutations. The second suspected ARAF isolate was confirmed to be susceptible to voriconazole (1 mg/L), itraconazole (0.5 mg/L), and posaconazole (0.25 mg/L).

## 4. Discussion

The extent and distribution of ARAF isolates in Africa remain largely unexplored, although arguments of their presence in Africa exist [7,8]. This study describes, for the first time, the presence of azole-resistance in the environment of *A. fumigatus* in Burkina Faso and in Western Africa with a prevalence of 2% among *A. fumigatus* positive samples (1/51). The prevalence of ARAF in Bobo-Dioulasso seems to be much lower than that reported in Tanzania (13.9%, 15/108) and Kenya (27%, 13/48) [5,6]. Both countries are located in East Africa and have intense farming with extensive use of azole-fungicides and substantial international trade, which can increase the risk of selection of resistant isolates and the import of ARAF from other countries.

This low prevalence of ARAF isolates in Bobo-Dioulasso could be explained by the persistence of traditional agricultural practices consisting of using plant waste at the end of each agricultural season to feed the animals. In addition, in some localities agricultural fields are burnt in order to prepare for the next agricultural season. These two traditional practices could prevent the development of conditions that favor the selection of ARAF isolates in Burkina Faso, such as plant waste stockpiling, which we could not find in the sites sampled for our study [13]. Furthermore, the low availability of data on the use of azole fungicides in agricultural areas in Burkina Faso could also explain the low prevalence of ARAF isolates observed in our study [8,14].

ARAF isolate identified was located an urban area and *A. fumigatus* positive samples were more frequently encountered with samples from urban areas (nursery, hospital, university garden, and residential). The intensive use of pesticides in agriculture fields in rural areas limits the proliferation of fungi. Besides, maintaining a certain level of humidity in urban sites favors the proliferation of *Aspergillus* species.

The MICs for voriconazole (MIC = 2 mg/L) fell under the area of technical uncertainty (ATU) suggested by the EUCAST committee in which voriconazole could be still used in clinical settings (non-invasive infections) if sufficient exposure is assured, but to our knowledge this is not performed in our screened region [15].

None of the most commonly described mutations among ARAF isolates (TR34/L98H and TR46/Y121F/T289A) were identified [3]. The *cyp51A* gene mutations observed in this study were F46Y/M172V/E427K. These mutations have been identified in both azole-susceptible and resistant *A. fumigatus* isolates in other studies [16,17]. The F46Y/M172V/E427K mutations are located at the periphery of the protein, a region predicted to neither interact with azole compounds nor to affect structural integrity [18]. This suggests that the found mutations might not be the molecular mechanism behind the observed resistance to azoles. Other mechanisms such as mutations of the *HapE* or *Hmg1* gene, overexpression of the *cyp51B* gene, efflux pumps, exogenous cholesterol import, or transcriptional rewiring with a loss of the cofactor 2 complex could be the cause of the ARAF isolate observed in our study [19,20,21,22,23,24]. On the other hand, in some studies the mechanism of resistance remains unidentified in 20% to 50% of cases [25] and might be the case for our ARAF isolate.

## 5. Conclusions

This study described for the first time the presence of *A. fumigatus* isolates resistant to an azole antifungal in the environment in Burkina Faso with a low prevalence of resistance. Our study supports the need to perform more extensive environmental studies that include other regions of Burkina Faso to confirm this observed azole-resistance prevalence and the need for azole-resistance surveillance in clinical settings.

## Figures and Tables

**Table 1:** Characteristics of *A. fumigatus* isolates by environmental site.

Sites	Number (N°) of Samples (%)	N° of *A. fumigatus* Positive Samples (%)	N° of *A. fumigatus* Isolates (%)	N° of Suspected ARAF Isolates	N° of Confirmed ARAF Isolates
Agriculture field (cotton, rice, market gardening)	50 (41.67)	8 (15.69)	229 (35.45)	0	0
Hospital	15 (12.5)	7 (13.72)	15 (2.32)	0	0
Residential	20 (16.67)	15 (29.41)	150 (23.22)	1	0
Forest	20 (16.67)	8 (15.69)	25 (3.87)	0	0
University garden	5 (4.17)	3 (5.88)	5 (0.77)	1	1
Nursery	10 (8.33)	10 (19.61)	222 (34.37)	0	0
Total	120 (100)	51 (100)	646 (100)	2	1
